# Nerve allograft histology after banking following complicated reconstruction

**DOI:** 10.1002/ccr3.3599

**Published:** 2020-12-09

**Authors:** David P. Foley, Cameron Cox, Joash Suryavanshi, Bradley Osemwengie, Brendan J. MacKay

**Affiliations:** ^1^ Department of Orthopedic Surgery and Rehabilitation Texas Tech Health Sciences Center Lubbock TX USA; ^2^ University Medical Center Lubbock Lubbock TX USA

**Keywords:** nerve allograft, nerve banking, nerve graft, nerve graft salvage, nerve histology, nerve surgery

## Abstract

Burial of nerve allografts in muscle tissue for later use is a novel technique with no prior literature discussions. This may be a safe and effective technique for short‐term preservation of nerve allograft removed from a prior reconstruction.

## Graphical abstract

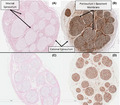

## 1 INTRODUCTION

Peripheral nerve injury is a frequent cause of motor and sensory dysfunction, occurring in as many as 3% of trauma patients.^1^, ^2^ If a small defect is present, the axonal ends may be anastomosed together with subsequent return of sensorimotor function. Larger defects must be bridged using grafts to maintain a tension‐free repair. While autograft usage is the gold standard for segmental nerve repair, inadequate expendable nerve tissue and donor site morbidity can preclude their use.^3^, ^4^ In these cases, allograft may be a viable reconstructive option. AxoGen's Avance^®^ is the only commercially available, FDA‐approved nerve allograft that has demonstrated clinical success.^3^ These cadaveric products undergo immunogenic processing, reducing the immune response of major histocompatibility complex‐mismatched allografts to that of an isograft.^5^, ^6^

Burial of nerve allografts within patient tissue for later use is a novel technique with no prior discussions in the literature. However, tissue banking is commonly used within medicine. For example, autologous skull segments are banked within the abdominal pocket after cranioplasty. This procedure is safe, inexpensive, sterile, and histocompatible.^7^ Subcutaneously preserved autologous cranioplasty has good long‐term outcomes, with 9% of banked grafts needing removal at 1‐year follow‐up.^8^ Further studies demonstrate decreased complications such as bone flap resorption when using autologous tissue banking vs cryopreservation.^9^

At our Level 1 Trauma Center, we are presented with complex cases including multiple extremity injuries that pose reconstructive challenges. At times, the best option for addressing these nerve injuries is the use of allografts. Unfortunately in trauma cases, even with careful staged reconstruction, complications such as adjacent tissue necrosis, vascular compromise, or infection may arise.

When presented with complications that necessitate takedown of the allograft neurorrhaphy, the graft must be removed from the original implantation site. Once the graft is unfrozen and at equilibrium with body fluid, removing it from the body induces cellular changes and degradation. Currently, no method exists for storing the graft extracorporeally. As a result, options are to remove the graft, discard it, and use a new graft for reconstruction or attempt to salvage these grafts. Considering the cost, limited availability of tissue resources, and frequency of these reconstructions, we determined it was worth investigating the viability attempting nerve allograft salvage.

## 2 CASE PRESENTATIONS

Two patients underwent nerve reconstruction using acellular nerve allograft (Avance^®^ Nerve Graft, AxoGen) with a later revision requiring takedown of the reconstructed nerve. The allograft was temporarily stored via burial in the patient's muscle.

### 2.1 Patient 1

A 28‐year‐old male sustained two gunshot wounds to the right arm causing open distal humerus and ulna fractures, as well as right ulnar nerve injury. He underwent ulnar nerve reconstruction using acellular nerve allograft. Five days post‐operatively, the patient had wound breakdown and muscle necrosis in the region of the reconstructed nerve requiring takedown of the allograft. At this time, the upper arm had not suffered necrosis and was considered a favorable tissue environment for banking. In an attempt to salvage the previously placed allograft, the graft was temporarily implanted within the triceps muscle through the previously closed incision in the upper arm. Two weeks later, the patient underwent ulnar nerve revision neurorrhaphy using the buried allograft. When we returned for reconstruction, the graft was cut to provide clean ends for anastomoses, shortening the graft. Additional graft was needed to complete the reconstruction, though less than would have been needed otherwise. The cut ends of the original graft were sent to an outside laboratory for histological evaluation. Three weeks post‐revision, he presented without complication. The patient was then lost to follow‐up.

### 2.2 Patient 2

Following a motor vehicle collision, a 19‐year‐old male underwent brachial plexus reconstruction with acellular allograft. Twelve days post‐operatively, the patient sustained an unsalvageable vascular complication and underwent transhumeral amputation. The graft was implanted into the pectoralis major muscle and later used for targeted muscle innervation (TMR) 2 days post‐amputation. TMR required less graft than the original reconstruction, and excess graft was sent for laboratory evaluation. While success of TMR cannot be definitively attributed to the viability of re‐implanted nerve allograft, it is worth noting that Patient 2 had no stump or phantom pain and was completely off of pain medication without complication at 3‐month follow‐up.

Using standard methodology, the banked nerves were stained using hematoxylin & eosin (H&E), laminin, and S100 staining. Unimplanted allograft samples were taken from lots matched to those of the buried nerves. These were also stained, and samples were evaluated for qualitative histological differences under light microscopy. H&E staining was used to evaluate the morphology and functional viability of the nervous tissue while laminin staining assessed the degree of Schwann cell proliferation and axonal regeneration. S100 was not performed on unimplanted allograft as these have been washed of all Schwann cells.

## 3 RESULTS

**FIGURE 1:**
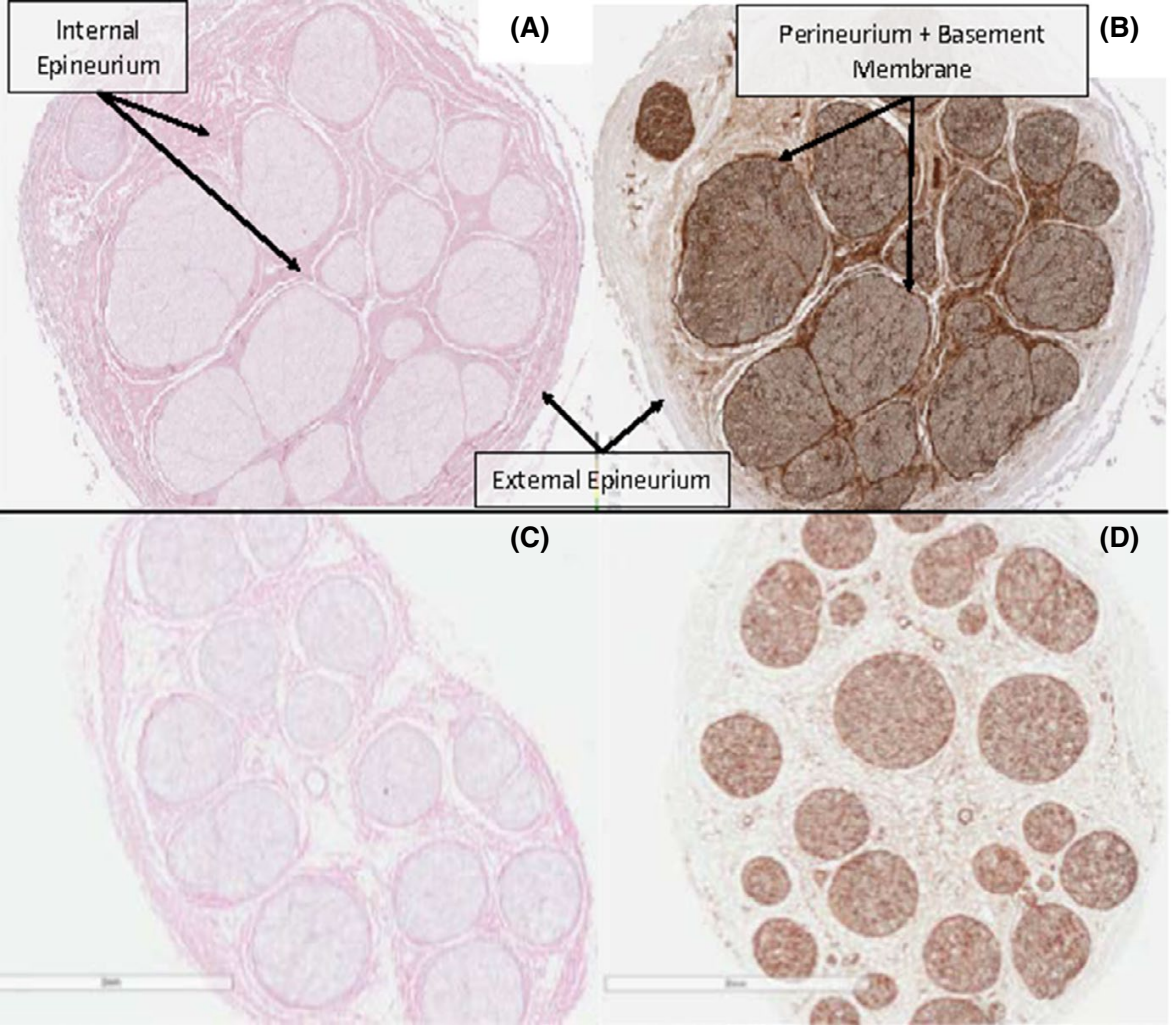
Histologic cross‐sectional slides showing ulnar buried samples (A,B), ulnar matched lot samples (C,D). H&E staining (A,C). Laminin staining (B,D). Connective tissues and basement membrane are denoted by arrows

**FIGURE 2:**
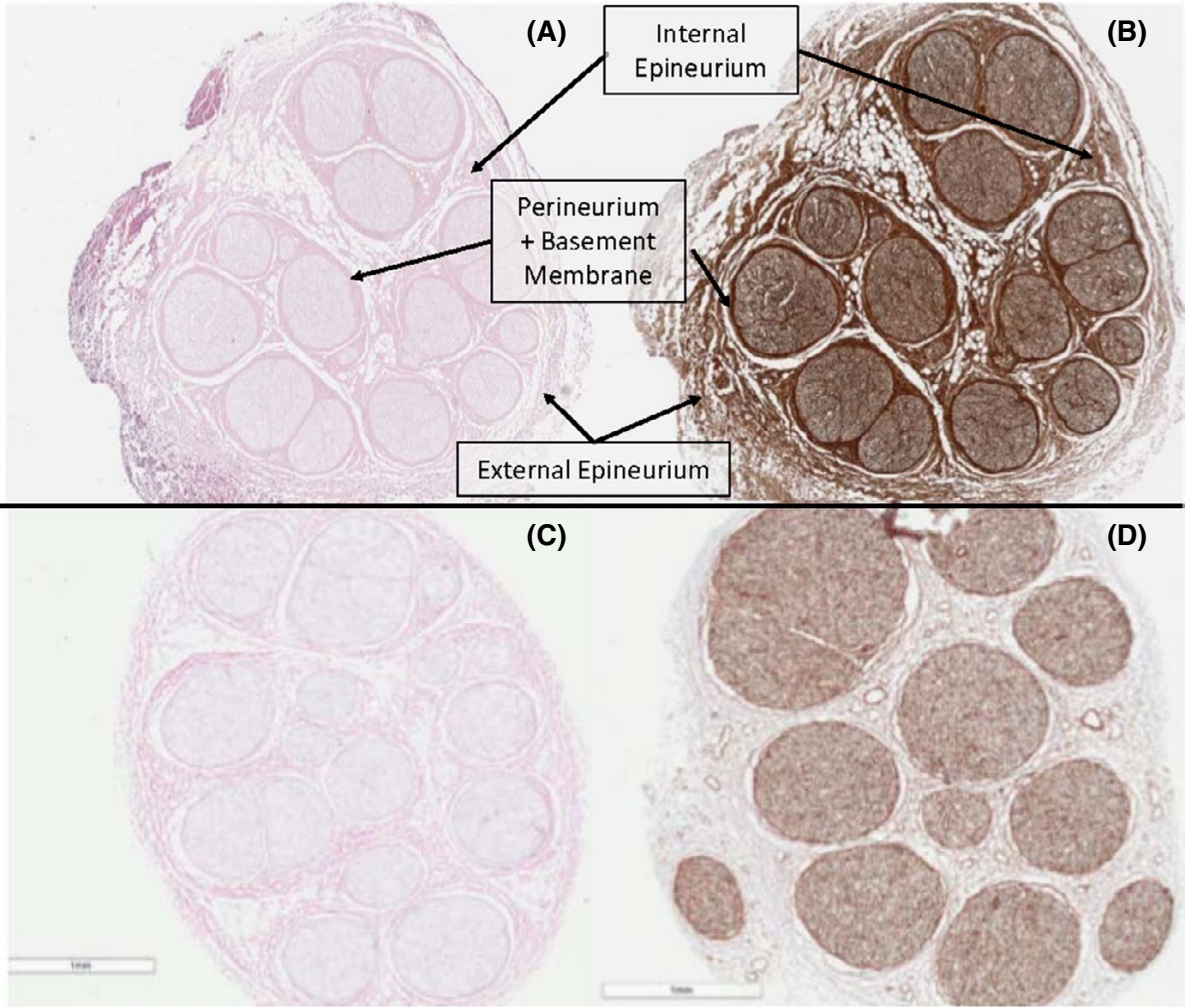
Histologic cross‐sectional slides showing brachial plexus buried samples (A,B), brachial plexus matched lot samples (C,D). H&E staining (A,C). Laminin staining (B,D). Connective tissues and basement membrane are denoted by arrows

H&E staining of allograft cross‐sections (Figures 1 and 2) exhibits increased eosin uptake in the interfascicular epineurium and perineurium of the buried ulnar allograft relative to a pre‐implanted allograft. This is consistent with collagen deposition and cytoplasmic debris within the epineurial connective tissue. Increased uptake of the stain is also seen in the perineurium of the buried brachial plexus allograft. Both buried allografts retained their compartmental architecture with parallel nerve fibers and lack axonal swelling.^10^ While eosin staining displays increases in connective tissue density, no gross morphological changes are present in the buried allografts. The lack of basophilic hematoxylin staining in the buried samples indicates minimal nuclear and RNA material is present in the buried sample and thus negligible infiltration by inflammatory leukocytes.^11^ The lack of macrophages typically presents in Wallerian degeneration and intact of nervous tissue structure suggests that burial of allografts in muscle tissue maintained viability of the nerves.

Laminin staining of post‐implantation samples indicates increased stain uptake relative to pre‐implantation allografts (Figure 1). As Schwann cells myelinate damaged nerves, they lay down extensive networks of basement membrane, encasing repaired nerve fibers. Laminins are critical components of basement membranes and are present in high amounts in early peripheral nerve regeneration. Our findings indicate widespread axonal repair and regeneration as recipient Schwann cells begin myelinating the allograft scaffolding. These findings are consistent with ongoing repair of an intact peripheral nerve and indicate that nervous tissue repair continued for the duration of the burial period. S100 staining confirmed that Schwann cells were already migrating into the buried sample.

## 4 DISCUSSION

Repair utilizing acellular nerve allografts is becoming a more widely accepted method of treating peripheral nerve deficits with data supporting equivalency between allograft and autograft for gaps up to 70 mm. Success of these allografts relies in part on surrounding muscle and soft tissue promoting graft revascularization and axonal regeneration. In complicated situations such as multisystem trauma or ballistics, surgeons attempt to optimize surrounding tissue before attempting peripheral nerve reconstruction. The reality of these situations is that even with careful staging, attempts to optimize the wound bed, and the appearance of healthy soft tissue, traumatic wounds are prone to complications. Adjacent soft tissue may become compromised, requiring allograft takedown. No data currently address management of previously placed allograft.

We hypothesized that burying the graft within patients’ intact muscle would preserve it for reuse within the same patient. Using laminin and H&E staining in conjunction for the examination of peripheral nerve viability and morphology, we found no clinically significant structural or histologic differences between lot‐matched, pre‐implanted allografts and post‐burial allografts. These findings suggest that nerve banking in muscle tissue may be safe and effective for the short‐term salvage/preservation of peripheral nerve allograft removed from a previous coaptation.

This paper is the first to histologically evaluate acellular peripheral nerve allograft after previous implantation and subsequent muscle burial in humans. Related studies assessed peripheral nerve histology following nerve burial in rats using electron microscopy to measure nerve fiber diameter, density, and myelin depth.^12^ In addition to electron microscopy, other studies have utilized toluidine blue stain for visualization of basophilic tissues, all within animal models.^3^, ^13^

Limitations of this report include a lack of serial allograft sampling, small sample size, and loss of Patient 1 to long‐term follow‐up. Buried allograft samples were not taken prior to initial implantation or nerve banking. This limits our comparisons to a similar nerve from the same lot. Histological sampling at the pre‐implantation, pre‐burial, and post‐burial stages would improve assessment of this technique. Clinical evaluations limited to 3‐month follow‐up are likely insufficient to establish long‐term outcomes in these cases.

Because this situation is relatively uncommon, it is likely that the sample size will never be large enough to conduct large‐scale analysis. Further validation of our findings might be possible if others encounter similar situations and replicate our process. The authors suggest that post‐implantation samples are obtained prior to allograft banking to allow for direct pre‐ and post‐burial comparison. Before reimplantation, resection of the graft edges from the previous coaptation site provides fresh ends for reconstruction. This shortens the graft, and additional graft may be needed. Future studies can use the quantitative methods described in prior studies and should track long‐term outcomes.

## 5 CONCLUSION

When unexpected surgical complications occur, allografts may potentially be preserved for reuse within the same patient through burial in muscle tissue. This technique avoids discarding viable nerve and reduces costs incurred by patients and healthcare providers. Its utilization could have a significant impact in Level 1 Trauma Centers where complex reconstructions and revisions are performed regularly.

## CONFLICT OF INTEREST

Though they are not directly funding this report, the authors would like to disclose the following support for BM: Paid teaching for TriMed. Paid teaching and consulting, as well as research support from AxoGen. Paid consulting for Baxter/Synovis and GLG. The remaining authors have nothing to disclose.

## AUTHOR CONTRIBUTIONS

DPF: involved in manuscript drafting, interpretation of data, and editing. CC: involved in manuscript drafting and editing. JS: involved in manuscript drafting and editing. BO: involved in manuscript drafting and editing. BJM: involved in critical revising, interpretation of data, and editing.

## ETHICAL APPROVAL

Statement of Informed Consent: All procedures followed were in accordance with the ethical standards of the responsible committee on human experimentation (institutional and national) and with the Helsinki Declaration of 1975, as revised in 2008. Appropriate consent has been obtained from all patients, prior to submission, in regards of the publication of images and data.

## Data Availability

All data presented and analyzed in this report are included in the published article.
